# Supplementary material for: Citizen scientists: Unveiling motivations and characteristics influencing initial and sustained participation in an agricultural project
Source: PLoS One. 2024 May 20;19(5):e0303103. doi: 10.1371/journal.pone.0303103 (PMC11104611; doi:10.1371/journal.pone.0303103)
Supplement: S1 Table — (DOCX) [file pone.0303103.s001.docx]

S1 Table: Factor analysis showing R-squared and factor loading for latent variables of Volunteer Factor Inventory (VFI) and intention and moral obligation to participate in the project.

|  | R-squared | Coefficient |
| --- | --- | --- |
| vfi_val1 | 0.3981 | 0.6309 |
| vfi_val2 | 0.6552 | 0.8094 |
| vfi_val3 | 0.6944 | 0.8333 |
| vfi_val4 | 0.3359 | 0.5796 |
| vfi_val5 | 0.2110 | 0.4593 |
| Overall | 0.8433 |  |
| vfi_und1 | 0.2888 | 0.5374 |
| vfi_und2 | 0.3341 | 0.5780 |
| vfi_und3 | 0.2641 | 0.5139 |
| vfi_und4 | 0.4667 | 0.6831 |
| vfi_und5 | 0.5109 | 0.7148 |
| Overall | 0.7403 |  |
| vfi_enh1 | 0.3971 | 0.6302 |
| vfi_enh2 | 0.6177 | 0.7860 |
| vfi_enh3 | 0.5710 | 0.7556 |
| vfi_enh4 | 0.6596 | 0.8122 |
| vfi_enh5 | 0.3304 | 0.5749 |
| Overall | 0.8579 |  |
| vfi_car1 | 0.5275 | 0.7263 |
| vfi_car2 | 0.4663 | 0.6829 |
| vfi_car3 | 0.7385 | 0.8593 |
| vfi_car4 | 0.6925 | 0.8322 |
| vfi_car5 | 0.7910 | 0.8893 |
| Overall | 0.9156 |  |
| vfi_soc1 | 0.5046 | 0.7104 |
| vfi_soc2 | 0.3817 | 0.6178 |
| vfi_soc3 | 0.5280 | 0.7266 |
| vfi_soc4 | 0.4209 | 0.6488 |
| vfi_soc5 | 0.5886 | 0.7672 |
| Overall | 0.8165 |  |
| vfi_prot1 | 0.5217 | 0.7223 |
| vfi_prot2 | 0.6120 | 0.7823 |
| vfi_prot3 | 0.3949 | 0.6285 |
| vfi_prot4 | 0.6551 | 0.8093 |
| vfi_prot5 | 0.8414 | 0.9173 |
| Overall | 0.9132 |  |
| tpbv_int1 | 0.6616 | 0.8134 |
| tpbv_int2 | 0.7119 | 0.8437 |
| tpbv_int3 | 0.4232 | 0.6505 |
| Overall | 0.8377 |  |
| tpbv_mobl1 | 0.4992 | 0.7066 |
| tpbv_mobl2 | 0.4690 | 0.6848 |
| tpbv_mobl3 | 0.2248 | 0.4741 |
| Overall | 0 .6846 |  |
