# Supplementary material for: Citizen scientists: Unveiling motivations and characteristics influencing initial and sustained participation in an agricultural project
Source: PLoS One. 2024 May 20;19(5):e0303103. doi: 10.1371/journal.pone.0303103 (PMC11104611; doi:10.1371/journal.pone.0303103)
Supplement: S2 Table — (DOCX) [file pone.0303103.s002.docx]

S2 Table: Factor analysis showing R-squared and factor loading for latent variables of General Values (GV).

|  | R-squared | coefficient |
| --- | --- | --- |
| gv_uni1 | 0.4070 | 0.6384 |
| gv_uni2 | 0.8450 | 0.9192 |
| gv_uni3 | 0.1594 | 0.3993 |
| gv_uni4 | 0.4041 | 0.6357 |
| Overall | 0.9913 |  |
| gv_welw1 | 0.6644 | 0.8151 |
| gv_welw2 | 0.4857 | 0.6969 |
| gv_welw3 | 0.4753 | 0.6894 |
| gv_welw4 | 0.3552 | 0.5960 |
| Overall | 0.8583 |  |
| gv_krac1 | 0.2407 | 0.4906 |
| gv_krac2 | 0.3180 | 0.5639 |
| gv_krac3 | 0.4571 | 0.6761 |
| gv_krac4 | 0.5452 | 0.7384 |
| Overall | 0.7385 |  |
| gv_zelf1 | 0.3173 | 0.5634 |
| gv_zelf2 | 0.1637 | 0.4046 |
| gv_zelf3 | 0.4854 | 0.6967 |
| gv_zelf4 | 0.2799 | 0.5291 |
| Overall | 0.6537 |  |
| gv_stim1 | 0.2920 | 0.5404 |
| gv_stim2 | 0.5198 | 0.7210 |
| gv_stim3 | 0.4901 | 0.7001 |
| gv_hedo1 | 0.2992 | 0.5470 |
| gv_hedo2 | 0.3253 | 0.5704 |
| Overall | 0.7582 |  |
| gv_trad1 | 0.1439 | 0.3794 |
| gv_trad2 | 0.3511 | 0.5926 |
| gv_trad3 | 0.3663 | 0.6052 |
| gv_trad4 | 0.1799 | 0.4241 |
| Overall \| | 0.6011 |  |
| gv_over1 | 0.5086 | 0.7131 |
| gv_over2 | 0.4340 | 0.6588 |
| gv_over3 | 0.3717 | 0.6097 |
| gv_over4 | 0.3569 | 0.5974 |
| Overall | 0.7467 |  |
| gv_veil1 | 0.2494 | 0.4994 |
| gv_veil2 | 0.5617 | 0.7495 |
| gv_veil3 | 0.4126 | 0.6423 |
| gv_veil4 | 0.3971 | 0.6301 |
| Overall | 0.7484 |  |
