# Supplementary material for: Citizen scientists: Unveiling motivations and characteristics influencing initial and sustained participation in an agricultural project
Source: PLoS One. 2024 May 20;19(5):e0303103. doi: 10.1371/journal.pone.0303103 (PMC11104611; doi:10.1371/journal.pone.0303103)
Supplement: S3 Table — (DOCX) [file pone.0303103.s003.docx]

S3 Table: Factor analysis showing R-squered and factor loading for latent variables of Environmental Concern (EC).

|  | R-squared | Coefficient |
| --- | --- | --- |
| ec_mil1 | 0.6778 | 0.8233 |
| ec_mil2 | 0.8658 | 0.9305 |
| ec_mil3 | 0.8048 | 0.8971 |
| ec_mil4 | 0.8353 | 0.9139 |
| Overall | 0.9467 |  |
| ec_mez1 | 0.7096 | 0.8424 |
| ec_mez2 | 0.4870 | 0.6979 |
| ec_mez3 | 0.4565 | 0.6757 |
| ec_mez4 | 0.6881 | 0.8295 |
| Overall | 0.8380 |  |
| ec_and1 | 0.5062 | 0.7115 |
| ec_and2 | 0.8198 | 0.9054 |
| ec_and3 | 0.6259 | 0.7911 |
| ec_and4 | 0.8296 | 0.9108 |
| Overall | 0.9951394 |  |
