# Supplementary material for: Citizen scientists: Unveiling motivations and characteristics influencing initial and sustained participation in an agricultural project
Source: PLoS One. 2024 May 20;19(5):e0303103. doi: 10.1371/journal.pone.0303103 (PMC11104611; doi:10.1371/journal.pone.0303103)
Supplement: S4 Table — Bold variables indicate a significantly higher answer for the group belonging to the bold variable. Effect size was calculated using Cohen’s d. VFI = Volunteer Function Inventory, EC = Environmental Concern, MI = Moral imperatives, SD = Socio-demographics, KN = Knowledge, GV = General Values. (DOCX) [file pone.0303103.s004.docx]

S4: Results of t-test between above average (sample size: 444) and below average (sample size: 365) contributors for all motivations and dispositional variables. Bold variables indicate a significantly higher answer for the group belonging to the bold variable. Effect size was calculated using Cohen’s d. VFI = Volunteer Function Inventory, EC = Environmental Concern, MI = Moral imperatives, SD = Socio-demographics, KN = Knowledge, GV = General Values..

|  |  | **Above*** | | **Below**** | |  | | | |
| --- | --- | --- | --- | --- | --- | --- | --- | --- | --- |
|  | *Variable* | *Mean* | *std. dev* | *Mean* | *std. dev* | *t-test* | *p-value* | *Cohen’s d* | *[90% conf. interval]* |
| *VFI* | Values | 5.31 | (0.93) | 5.35 | (0.90) | -0.71 | 0.48 | 0.05 | -0.09 0.19 |
|  | Understanding | 5.01 | (0.99) | 4.99 | (1.02) | -0.21 | 0.83 | 0.02 | -0.15 0.12 |
|  | Enhancement | 4.36 | (1.21) | 4.41 | (1.14) | 0.63 | 0.62 | 0.03 | -0.10 0.17 |
|  | Career | 2.83 | (1.40) | **2.99** | **(1.43)** | 1.62 | 0.05 | 0.11 | -0.02 0.25 |
|  | Social | 3.60 | (1.21) | 3.65 | (1.17) | 0.58 | 0.56 | 0.04 | -0.10 0.18 |
|  | Protective | 2.81 | (1.33) | 2.81 | (1.27) | 0.10 | 0.92 | 0.00 | -0.13 0.15 |
| *EC* | Nature | **6.11** | **(0.90)** | 5.98 | (0.97) | -1.95 | 0.03 | -0.14 | -0.28 0.00 |
|  | Myself | 4.89 | (1.16) | 5.00 | (1.18) | 1.51 | 0.13 | 0.11 | -0.03 0.25 |
|  | Others | **5.83** | **(0.99)** | 5.73 | (1.02) | -1.35 | 0.09 | -0.10 | -0.23 0.04 |
| *MI* | Feeling of responsibility | 5.98 | (1.78) | 6.12 | (1.82) | 1.05 | 0.29 | 0.07 | -0.06 0.21 |
|  | Moral obligation | **4.27** | **(1.23)** | 3.94 | (1.14) | -3.86 | 0.00 | -0.27 | -0.41 -0.13 |
| *SD* | Age | **55.35** | **(14.22)** | 48.45 | (13.86) | -6.94 | 0.00 | -0.49 | -0.63 -0.35 |
| *KN* | Objective | **9.06** | **(2.81)** | 8.48 | (3.00) | -2.82 | 0.00 | -0.20 | -0.34 -0.06 |
|  | Subjective | 10.00 | (3.46) | 9.91 | (3.69) | -0.35 | 0.71 | -0.03 | -0.16 0.11 |
| *GV* | Universalism | **5.73** | **(0.67)** | 5.62 | (0.70) | -2.26 | 0.01 | -0.16 | -0.30 -0.02 |
|  | Benevolence | 5.80 | (0.71) | 5.82 | (0.70) | 0.38 | 0.70 | 0.03 | -0.11 0.17 |
|  | Power | 3.43 | (1.00) | 3.43 | (1.00) | -0.06 | 0.95 | 0.00 | -0.14 0.13 |
|  | Achievement | 4.50 | (0.92) | 4.50 | (0.96) | 0.05 | 0.95 | 0.00 | -0.13 0.14 |
|  | Self-direction | 5.53 | (0.70) | 5.54 | (0.75) | 0.16 | 0.87 | 0.01 | -0.13 0.15 |
|  | Stimulation | 5.09 | (0.80) | 5.19 | (0.84) | 1.71 | 0.04 | 0.12 | -0.02 0.26 |
|  | Tradition | 5.14 | (0.77) | 5.11 | (0.75) | -0.46 | 0.64 | -0.16 | -0.30 -0.02 |
|  | Conformity | **5.18** | **(0.85)** | 5.09 | (0.95) | -1.49 | 0.07 | -0.11 | -0.24 0.03 |
|  | Security | **4.96** | **(0.97)** | 4.85 | (1.04) | -1.58 | 0.06 | -0.11 | -0.25 0.03 |
|  | Participation intention | **4.61** | **(0.58)** | 4.52 | (0.62) | -2.00 | 0.02 | -0.14 | -0.28 0.00 |
| ***Above: participants who filled in more than the average amount of data during the project , **Below: participants who filled in less than the average amount of data during the project.** | | | | | | | | | |
